# Translational Strategies to Eliminate Chronic Hepatitis B in Children: Prophylaxis and Management in East Asian Countries

**DOI:** 10.3389/fped.2021.809838

**Published:** 2022-02-04

**Authors:** Ben Kang, Dae Yong Yi, Byung-Ho Choe

**Affiliations:** ^1^Department of Pediatrics, School of Medicine, Kyungpook National University, Daegu, South Korea; ^2^Department of Pediatrics, College of Medicine, Chung-Ang University Hospital, Chung-Ang University, Seoul, South Korea

**Keywords:** viral replication, hepatocytes, turnover, cccDNA, hepatitis B virus, nucleos(t)ide analog

## Abstract

Translational medical research on hepatitis B virus (HBV) infection and chronic hepatitis B (CHB) pathogenesis provides guidance on strengthening the treatment and prevention strategies of CHB. Preventing vertical transmission is the key to eliminating HBV infection in children. The understanding of HBV replication, hepatocyte turnover, and the fate of covalently closed circular DNA (cccDNA) would help establish a personalized application of the guidelines, especially concerning the discontinuation of nucleos(t)ide analog (NA) treatment in children. Transplacental leakage of HBV-infected maternal blood is suggested as the leading cause of vertical transmission. Prenatal maternal prophylaxis could diminish maternal HBV viremia at delivery, to reduce the risk of neonatal HBV infection. The meaning of the expression “no additional risk of breast milk feeding” is thereby explained. Understanding the untreated natural course of CHB in children and the course changeable by treatment is important to apply individualistic strategies and avoid the immoral selection of treatment indications. NAs with potent efficacy and a high barrier to drug resistance should be used as first-line treatment to reduce the likelihood of NA-resistant HBV development because the rate of mutant HBV emergence might count on the infected hepatocyte turnover rate in chronic HBV infection. Although elimination of intranuclear cccDNA is difficult by NAs alone, a cure is possible by human immunity and hepatocyte turnover. The reduction of intranuclear cccDNA occurs after the destruction of HBV-infected hepatocytes, non-cytolytic immune response, apoptosis of hepatocytes, and compensatory cell proliferation. Therefore, consolidation therapy after NA-induced hepatitis B e-antigen seroconversion must be necessary for a sufficient period. This review also summarizes the treatment strategies of CHB in children based on the practical application of translational research.

## Introduction

The risk of developing hepatitis B virus (HBV)-related hepatocellular carcinoma (HCC) in children in Asian countries is higher than in children in western countries (1, 2). The most common HBV genotypes are C, followed by B, in northeastern China (3). In Korea and Japan, the prevailing genotype is C (4). In Taiwan, where genotype B is most prevalent, genotype B is predominant in HCC of patients <35 years, including children, and hepatitis B e-antigen (HBeAg) seroconversion in genotype B occurs at a younger age than in genotype C (5).

**Figure 1:**
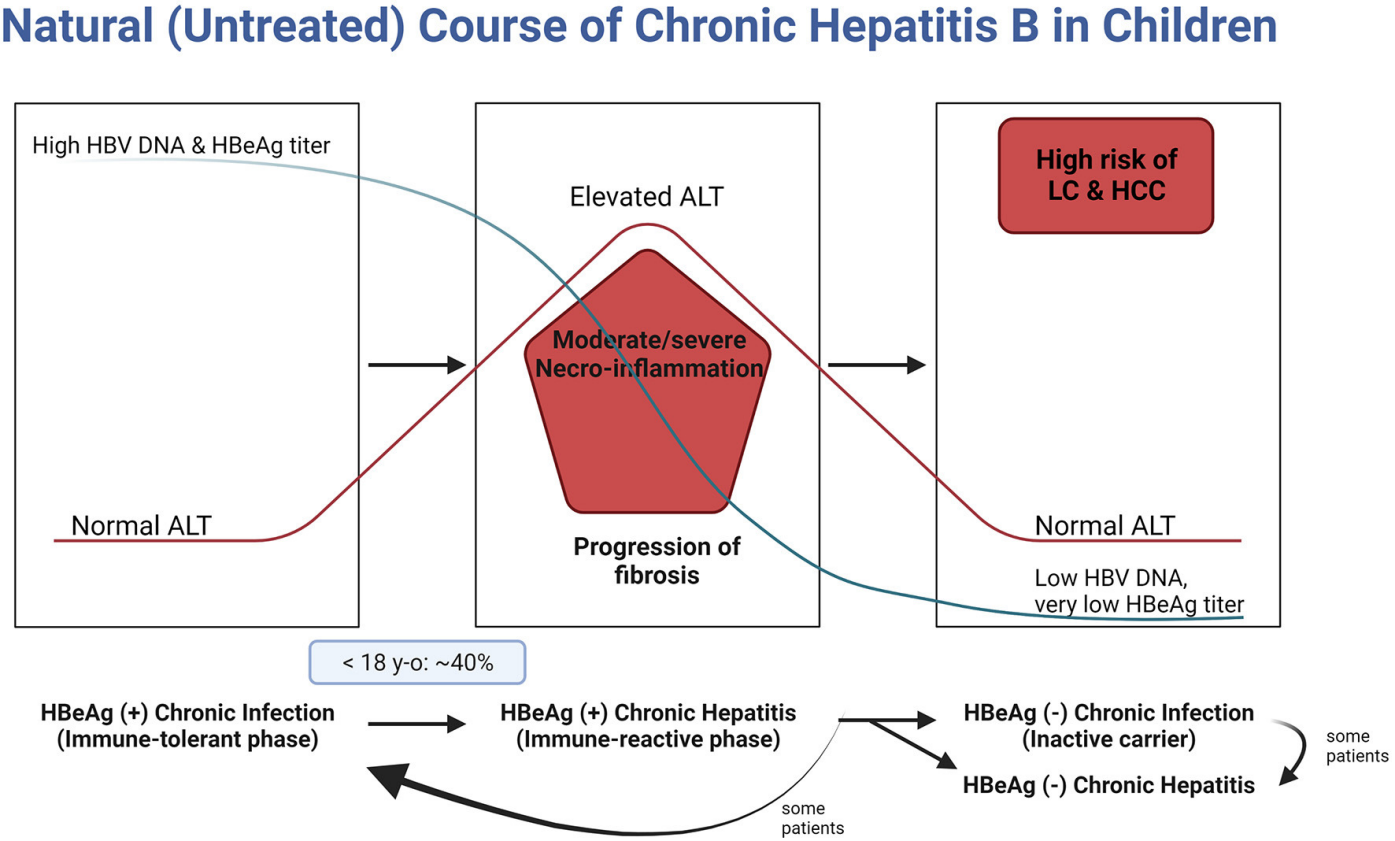
Natural course of chronic hepatitis B in children. HBV, hepatitis B virus; DNA, deoxyribonucleic acid; HBeAg, hepatitis B envelope antigen; ALT, alanine aminotransferase; LC, liver cirrhosis; HCC, hepatocellular carcinoma.

After vertical HBV infection, 90% of infants become HBV carriers if postnatal prophylaxis is not done. The initial phase of natural course of chronic hepatitis B (CHB) is the immune-tolerance phase (HBeAg-positive chronic infection), in which serum alanine aminotransferase (ALT) remains within normal range though the serum HBV DNA level is over 20,000 IU/ml (nearly the upper limit of the measurement in practice) due to the active HBV replication rate; however, liver damage is minimal (6). As the phase enters the immune-clearance phase (HBeAg-positive chronic hepatitis, immune-reactive phase, or so-called active hepatitis), ALT rises accompanied by necrosis and fibrosis of liver tissues. Though untreated during the phase of active hepatitis, the phase of CHB transit to the non/low-replicative phase by the natural course of CHB (6, 7) (Figure 1). Moderate to severe hepatitis for a prolonged period during active hepatitis can lead to the development of HCC even in childhood (8). In many children with untreated active hepatitis, elevated ALT is normalized, and liver pathology can be minimal. However, spontaneous HBeAg seroconversion in children does not necessarily lead to a good prognosis, and in some children, liver cirrhosis (LC) or even HCC may occur (9). It is essential to minimize the severity of necro-inflammation during active hepatitis and reduce the length of the HBeAg-positive chronic hepatitis, through timely and appropriate treatment without negligence (10) (Figure 1).

Therefore, two major issues need to be reviewed when considering HBV infection and CHB pathogenesis. The first is how to prevent vertical transmission to eliminate CHB in children, and the second is when to stop the nucleos(t)ide analog (NA) treatment in children to prevent the relapse of CHB. To make appropriate health decisions, understanding pathogenesis and translational medical research is essential. This review focuses on preventing and managing CHB in terms of HBV transmission, replication, and hepatocyte turnover.

## Vertical Transmission of HBV

The primary transmission route of HBV is perinatal, especially during delivery in endemic areas (11). Thus, universal vaccination and maternal screening for hepatitis B surface antigen (HBsAg) are essential to decrease the prevalence of and complications associated with CHB. Thanks to universal vaccination and postnatal prophylaxis, the HBsAg-positive population markedly declined in younger age groups between 1998 and 2010 in South Korea. The prevalence decreased from 2.2% in 1998 to 0.1% in 2010 among adolescents (10–19 years) (12). Therefore, HBsAg seropositivity in child-bearing ages (assumed as 25–34-year-old Korean women) is expected to be 0.1% in 2025, which means the prevalence of HBsAg-positive newborn babies is expected to be <0.01% in 2025.

However, in some studies, current immunoprophylactic measures inadequately protect 18% of newborn infants born to HBeAg-positive mothers (13). Prophylaxis failure was almost 5% and increased to 7.6% if maternal HBV DNA levels were >8 log_10_ copies/ml. High levels of maternal HBV DNA and detectable viral DNA in the cord blood were regarded as risk factors for prophylaxis failure (14). If either threatened abortion or threatened preterm labor (or both) has occurred during pregnancy, transplacental microtransfusion during delivery or intrauterine transmission is assumed to be the route of vertical transmission (15). Therefore, prolonged labor and/or threatened preterm abortion are associated with a high risk of vertical transmission.

### Antepartum Transmission

Intrauterine HBV infection occurs when the placental barrier becomes infected. In 1987, Lin et al. suggested that the transplacental leakage of HBeAg-positive maternal blood is the most likely cause of intrauterine HBV infection (15). This is thought to be induced by uterine contractions during pregnancy and the disruption of placental barriers, such as in threatened abortion and/or threatened preterm labor (15). HBV infection of the placenta has been reported to be markedly correlated with HBV DNA in the cord blood (16).

Postnatal prophylaxis failures occur almost exclusively in HBeAg-positive women with high HBV DNA levels. NAs, such as lamivudine, and the preferred agent, tenofovir (TDF), may be administered in the third trimester to inhibit viral replication in HBsAg-positive pregnant females with high HBV DNA loads (>200,000 IU/ml) (Table 1) (6). This prenatal prophylaxis significantly diminishes maternal HBV viremia at delivery, thereby reducing the risk of vertical transmission via placental microtransfusion compared to conventional prophylaxis (13). Breast milk feeding would be safe. Neonatal TDF plasma concentrations were extremely low by simulation, so that it is unlikely to produce adverse events or select for mutant HBV (17).

**Table 1:** Prenatal and postnatal prophylaxis.

	**Antenatal prophylaxis**	**Postnatal prophylaxis**
Medication	TDF	HBIG + HBV vaccine
Timing	Up to 3 months before the delivery of the baby	within 12 h after delivery

*TDF, tenofovir disoproxil fumarate; HBIG, hepatitis B immune globulin; HBV, hepatitis B virus*.

### Intrapartum Transmission

HBsAg and HBV cannot cross the placenta, whereas HBeAg can (18). However, trauma during labor may result in maternal–fetal blood microtransfusion. Uterine contractions can cause microdamage to the placenta, which may result in placental leakage (19). Regarding the risk of maternal–fetal microtransfusion, the duration of uterine contractions would be shorter in elective cesarean section (ECS) cases than urgent cesarean section (UCS) cases or prolonged spontaneous vaginal delivery (11). The rate of vertical transmission of HBV infection to infants delivered by ECS is significantly lower than in those delivered vaginally or by UCS. ECS for HBeAg-positive mothers with pre-delivery levels of HBV DNA ≥1,000,000 copies/ml could reduce the risk of vertical transmission (20).

### Postpartum Transmission

Postnatal prophylaxis with hepatitis B immune globulin (HBIG) and HBV vaccination is important before the HBV makes its way to the liver and starts replicating (Table 1). However, regarding the risk of breastfeeding, the rate of HBV vertical transmission in the HBV DNA-positive breastfed group was not significantly different from the formula milk-fed group in a study conducted by Yin et al. in 2013 (21). Furthermore, in a recent Chinese study, prophylaxis failure was higher in formula-fed infants than in breastfed infants regardless of HBV DNA levels in breast milk (21). Thus, infection is assumed to occur by placental leakage during or before delivery, not by breastfeeding.

The HBV load in breast milk is very low. Even if the baby swallows blood through the cracked nipples, HBV would be diluted in the breast milk. HBV transmission from breast milk to the gastrointestinal mucosa is not like blood to intravascular transmission. There is no evidence that HBV can directly infect gastrointestinal mucosal cells (11).

In brief, babies with minimal infection during delivery are protected by HBIG plus HBV vaccine, which should be given within 12 h after delivery. Despite postnatal prophylaxis, babies infected by high viral load before delivery may not be protected without prenatal maternal prophylaxis. In other words, there would be no additional risk by breastfeeding if conventional postnatal prophylaxis was successfully performed.

## Translational Strategy for HBV Clearance

### HBV Replication

**Figure 2:**
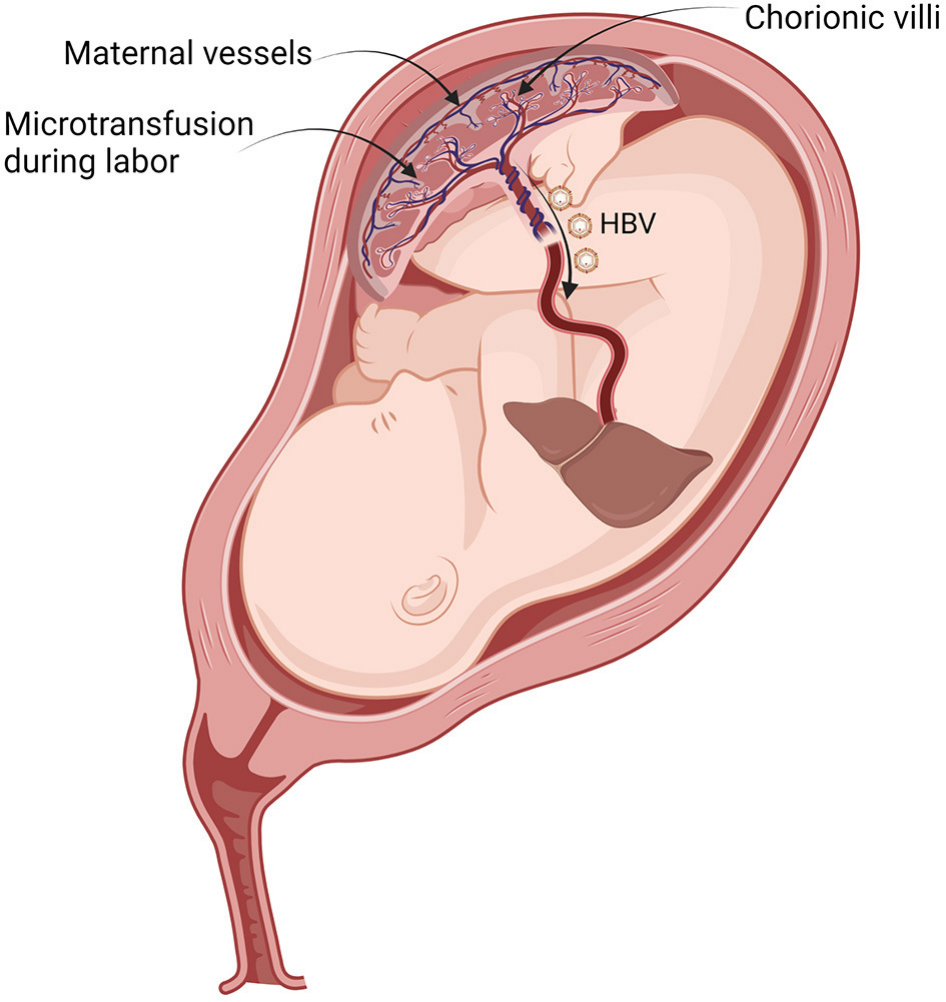
Vertical transmission of HBV during labor contraction. By placental leakage, transplacental maternal–fetal microtransfused blood containing HBV flows directly into the neonatal liver. HBV, hepatitis B virus.

After maternal–fetal microtransfusion occurs during delivery, HBVs flow directly into the neonatal liver without immunologic blockade and start to replicate exponentially in hepatocytes (Figure 2). Intravenously inoculated HBV immediately passes through the fenestrations from the sinusoids of the liver into the space of Dissé adjacent to hepatocytes. Hepatic sinusoidal cells function as a portal of entry for HBV (22). HBV entrance into the newborn's hepatocytes is the target for the protective immune response of HBIG (23).

The viral envelope of HBV attaches to hepatocytes, releasing the viral nucleocapsid into the cytoplasm. The released nucleocapsids are then transported to the nucleus, following which the formation of covalently closed circular DNA (cccDNA) ensues (22). The subsequent processing involves RNA transcription using cccDNA as an intranuclear template, packaging of pregenomic RNA and viral polymerase (Pol) in immature nucleocapsids in the cytoplasm of hepatocytes, and production of the progeny relaxed circular viral DNA genome by reverse transcription within immature nucleocapsids.

HBV replicates rapidly with minimally estimated doubling times ranging between 2.2 and 5.8 days (mean, 3.7 ± 1.5 days). Peak HBV production rate is estimated to be at least 10^13^ virions/day and a maximum production rate of an infected hepatocyte of 200 to 1,000 virions/day on average. At this peak rate of virion production, mutations would be created on each day (24). After a peak viral load in serum of ~10^10^ HBV DNA copies/ml is attained, the clearance of HBV DNA follows an initial rapid decline characterized by a mean half-life (t_1/2_) of 3.7 ± 1.2 days. Finally, viral clearance occurs at a variable rate (t_1/2_ of 4.8–284 days) and may relate to the rate of loss of infected hepatocytes (24). The rate of emergence might depend on the rate of infected hepatocyte turnover in chronic HBV infection (25). Therefore, NAs with potent efficacy and a high barrier to drug resistance should be used as the first-line treatment to reduce the likelihood of mutant HBV development. Unfortunately, replication resumes frequently when treatment is withdrawn earlier than the guideline recommendations.

### Nuclear cccDNA

Chronic HBV infection is maintained by cccDNA, the template of RNA transcription and replication by viral Pol, and reverse transcription. In quiescent hepatocytes, cccDNA is a stable molecule that can persist throughout the life span of hepatocytes. However, immune-mediated cell injury and compensatory hepatocyte proliferation may favor the decrease in cccDNA and the selection of cccDNA-free cells (26). Allweiss et al. reported that cccDNA reduction is mainly the consequence of hepatocyte division in immunocompetent hosts with inflammation, but the effect of cytokines seems to be minimal (27).

**Figure 3:**
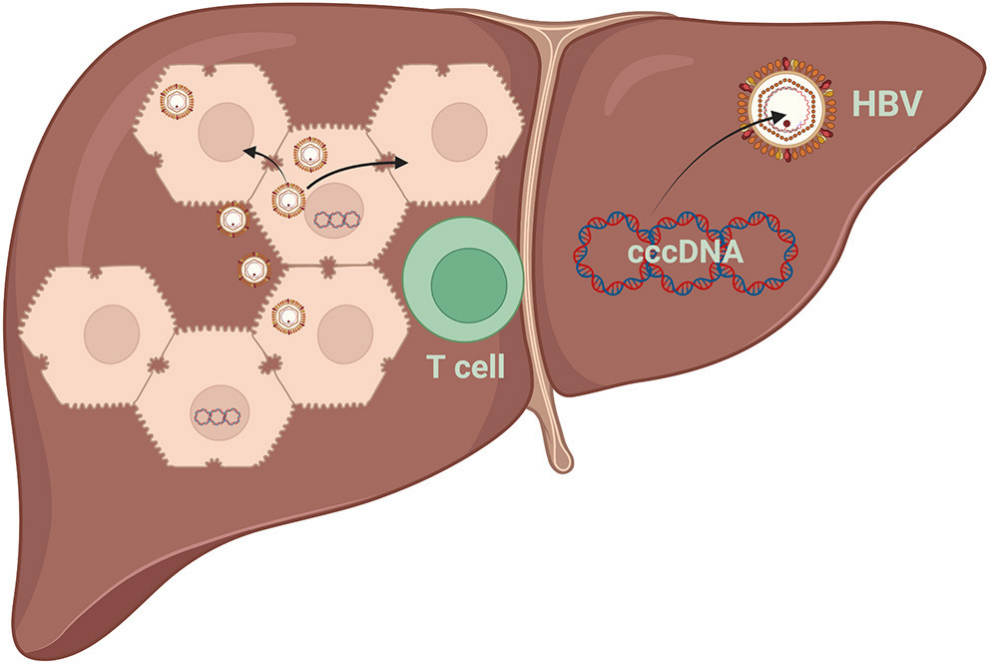
Destruction of HBV-infected hepatocytes results in reduced intrahepatic cccDNA levels. Apoptosis of hepatocytes and compensatory cell proliferation enhance the reduction of intrahepatic cccDNA loads, even without a cytolytic immune response. HBV, hepatitis B virus; cccDNA, covalently closed circular DNA.

Infection and reinfection of cured and/or naive hepatocytes may be crucial in infection persistence (28). cccDNA amplification predominantly depends on an extracellular route of receptor-mediated infection (29). Persistently infected hepatocytes allow viral spreading between hepatocytes once cell proliferation ends and when the inhibition of dissemination is uncontrolled (Figure 3) (27).

The persistence of cccDNA results in the failure of viral clearance and relapse of viral activity after stopping antiviral therapy with Pol inhibitors in patients with chronic HBV infection (30). Moreover, if viral suppression is insufficient, the selection of resistant mutants may occur (31). Although HBV-Pol inhibitors do not directly affect cccDNA, the emergence of fewer nucleocapsids into the pool due to the inhibition of viral DNA synthesis in the cytoplasm may explain the decrease in cccDNA levels. Long-term antiviral therapy will be required to adequately control HBV replication considering the longevity of cccDNA (30). The life span of cccDNA is 61 [36–236] days during HBeAg-positive infection (32).

There is a parallel decrease in serum HBsAg and intrahepatic cccDNA levels (33). Accordingly, cccDNA clearance is achieved through hepatocyte death. Moreover, the regeneration of infected hepatocytes can occur without cell death. Furthermore, NAs block new rounds of infection, which induces drastic destabilization and significant clearance of the cccDNA pool *in vivo* (26).

### Hepatocyte Turnover

Liver cells are differentiated cells with a very slow turnover in the healthy state of the adult liver. The normal rate of cell turnover in the liver is uncertain, but the average life span of hepatocytes is 5 months (34), and the functional life span of hepatocytes ranges from 200 to 400 days in mice (35).

In active hepatitis, hepatocytes can quickly enter the cell cycle and divide to compensate for the death of other hepatocytes. Therefore, it has been proposed that cell division may favor the dilution of cccDNA, so that cccDNA-free cells can be generated, whereas infected cells are forced to divide to compensate for the immuno-mediated loss of other infected cells (Figure 3) (36, 37).

### HBV Elimination

A cytotoxic T-lymphocyte (CTL) response is responsible for liver injury and viral clearance during HBV infection (38). HBV can be eliminated from hepatocytes by non-cytopathic mechanisms that are initiated through the action of cytokines induced during the inflammatory response. Non-cytopathic mechanisms of virus clearance from the liver and the possible CTL-mediated killing of infected hepatocytes are the primary mechanisms for virus elimination (Figure 3) (39).

The division of primary human hepatocytes, even without cytolytic mechanisms, could deplete substantial cccDNA loss (27). ALT flares reflect the destruction of HBV-infected hepatocytes and result in reduced intrahepatic cccDNA levels (40). Compensatory cell proliferation enhances the reduction of intrahepatic cccDNA loads and circulating antigen levels. However, a reservoir for HBV reinfection lies within a few persistently infected cells (27).

## Practical Issues Regarding HBV Treatment With Respect to HBV Replication and Hepatocyte Turnover

### Medications Approved for Children With CHB

Long-term NA treatment is more effective than interferon treatment in younger children (aged <7 years) (41). Furthermore, younger children (26.5% out of HBeAg-seroconverted patients) achieved a higher rate of HBsAg clearance (42).

Although antiviral treatment is never recommended in the immune-tolerant phase in children owing to no therapeutic effect and the development of NA resistance, the likelihood of HBsAg loss was higher if NA + α-interferon treatment was used (43). This result could be presumed as interferon-induced activation of T-cell immunity after NA-induced decrease in viral load. The favorable patient group would be young children with remaining thymus before major involution. Genotype differences might also explain these promising results.

TDF was approved by the Food and Drug Administration for children aged ≥12 years in 2012, and entecavir (ETV) was approved for children aged >2 years in 2014 (Table 2). TDF and tenofovir alafenamide (TAF) are currently under clinical trial for usage in children between 2 and 12 years. Long-term TDF treatment of CHB in children was effective without resistance (44).

**Table 2:** Comparison of treatment regimens for chronic hepatitis B in children.

	**Age (years)**	**Weight or BSA**	**Antiviral potency**	**Genetic barrier**	**Pharmacologic category**	**Monitoring parameters**
TDF	≥2	>10 kg	High	High	Nucleotide reverse transcriptase inhibitor	HBV DNA, ALT, HBeAg, anti-HBe Ab, HBsAg, renal function, lactic acid levels, serum electrolytes, anion gap, phosphate, 25-OH-Vitamin D levels, lipid profiles, urinalysis
ETV	≥2	>10 kg	High	High	Nucleoside reverse transcriptase inhibitor	HBV DNA, ALT, HBeAg, anti-HBe Ab, HBsAg, renal function, lactic acid levels
PegIFNα2a, PegIFNα2b	≥3	180 μg/1.73 m^2^	Moderate	High	Pegylated interferon α	CBC, liver function, renal function, TSH, uric acid

*BSA, body surface area; TDF, tenofovir disoproxil fumarate; ETV, entecavir; PegIFNα, pegylated interferon alpha; HBV, hepatitis B virus; DNA, deoxyribonucleic acid; ALT, alanine aminotransferase; HBeAg, hepatitis B envelope antigen; anti-HBe Ab, anti-hepatitis B envelope antibody; HBsAg, hepatitis B surface antigen; 25-OH-Vitamin D, 25-hydroxyvitamin D; CBC, complete blood count; TSH, thyroid stimulating hormone*.

### Whom to Treat

Indications for treatment in Korean children with CHB include HBV DNA levels >20,000 IU/ml, sustained elevation of serum ALT levels exceeding twofold of the upper limit of normal (ULN) levels for 6 months, and/or active hepatitis demonstrated in liver biopsies (45). There should be obvious evidence that the ALT elevation is associated with CHB. Asian Pacific guidelines recommend an observation period of 12 months for the possible occurrence of spontaneous HBeAg seroconversion in HBeAg-positive children with ALT levels >1 × ULN levels (Table 3) (46).

**Table 3:** Indications of treatment in children with HBeAg-positive chronic hepatitis B according to the guidelines of APASL and KASL.

	**ALT levels**	**HBV DNA levels**	**Liver disease severity**	**Treatment consideration if**
APASL	Elevated levels	High values	Necro-inflammation and fibrosis by liver biopsy, fibrosis by Fibroscan	>1 × UNL: observe for 12 months
KASL	≥2 x UNL	≥20,000 IU/ml	Liver biopsy	≥2 × UNL: observe for at least 6 months or consider liver biopsy for earlier treatment

*HBeAg, hepatitis B envelope antigen; APASL, Asian Pacific Association for the Study of the Liver; KASL, Korean Association for the Study of the Liver; ALT, alanine aminotransferase; HBV, hepatitis B virus; DNA, deoxyribonucleic acid; UNL, upper normal limit*.

The clearance of infected hepatocytes from the liver correlates with hepatocyte turnover. Therefore, in the absence of the accelerated elimination of infected hepatocytes (immune-clearance phase), inhibitors (NAs) of virus replication are required for a lifelong period to substantially reduce the burden of infected hepatocytes in the liver (47). Therefore, it is difficult to treat CHB if antiviral treatment (NA) is initiated in the immune-tolerant phase without activated T-cell immunity.

### When to Start Treatment

It is impossible to predict the duration of the immune-tolerant phase. It can even exceed three decades in patients vertically infected by HBeAg-positive mothers (46). In addition, it remains unclear which infected individuals would enter the immune-clearance phase by the activation of T-cell immunity. Therefore, it is important to monitor children and adolescents until they become adults to check whether there is an elevation in their ALT levels (Figure 1).

In Korea, where HBV genotype C is predominant, vertically and chronically infected patients usually experience a longer period of active HBV replication, resulting in delayed HBeAg seroconversion (48). Therefore, they are at risk for more severe fibrosis, cirrhosis, and even HCC than patients infected with HBV genotype B (49).

About 90% of children are HBeAg-positive by ages 10–15 years (50). Meanwhile, the proportion of children with CHB entering the initial period of the immune-clearance phase differs according to age. For example, this proportion in Korea was 11.7% and 39.7% in children ages <12 and <18 years, respectively (Figure 1) (51). Nevertheless, if the active immune phase occurs in young children, the likelihood of NA-induced HBeAg and HBsAg seroconversion is much higher than in adults (41).

Pretreatment of low HBV DNA load and/or high ALT levels is associated with favorable prognostic factors in the treatment of chronic hepatitis. Low HBV DNA load and/or high ALT levels are observed during the late period of the immune-clearance phase. Therefore, the efficacy of antiviral treatment would be much better during imminent spontaneous HBeAg seroconversion than during the early period of the immune-clearance phase despite delayed treatment (10). Timely treatment in the immune-clearance phase is crucial as delayed or no treatment may increase the incidence of LC or HCC (9, 10). Antiviral treatment is also needed for patients with low HBV DNA load and/or high ALT levels because the liver damage would worsen without treatment. Furthermore, the immune-tolerant phase can occur again in children if not treated (6).

### What to Monitor During Treatment

Once antiviral treatment is initiated, ALT normalization, undetectable serum HBV DNA load, and serum HBeAg loss (or seroconversion) are the primary treatment goals. In the real world, the shorter duration of HBeAg positivity during treatment may predict how early the primary goal will be achieved and the higher rate of HBV DNA and HBeAg titer decrease. In addition, monitoring of medication compliance of antiviral agents is very important to reduce the development of antiviral resistance during the entire treatment duration. Selective pressure from NA treatment may result in mutant HBV disseminating to other hepatocytes and replacing wild type HBV (28). Sufficient maintenance of NA treatment during consolidation therapy after HBeAg clearance is also important, especially for patients with prolonged HBeAg positivity before HBeAg clearance. Maintaining complete virologic response and HBeAg seroconversion during consolidation therapy could predict the low chance of virologic relapse after NA treatment discontinuation. Further, if HBsAg titer rapidly decreases in younger children, consolidation therapy could be extended to achieve HBsAg clearance (42).

Due to the previous injury to hepatocytes, the fibrotic burden might be responsible for the residual risk of HCC (52). As HCC and LC can develop even in children, alpha-fetoprotein (AFP), liver ultrasonography, and FibroScan may be helpful, especially for children with a family history of HCC or LC. Regular follow-ups are necessary for patients who have experienced a severe course of active hepatitis (immune-clearance phase), relapse after NA treatment discontinuation, or prolonged breakthrough despite NA treatment.

### When to Stop Treatment With Antiviral Agents

How long should the antiviral agent (TDF/ETV) be used? This depends on the pretreatment condition of the patient (such as ALT levels, HBV DNA load, and age), therapeutic response (in terms of the decrease rate in HBV DNA load and HBeAg titer), and treatment compliance to decrease the development of antiviral resistance of remaining intrahepatic HBV.

It is important to prevent relapse after completing HBeAg seroconversion by continuing treatment for at least 12–36 months; preferably, additional therapy should be continued until 3 years after HBeAg/anti-HBe seroconversion (46). In addition, investigators have suggested that NAs should be continued for at least 2–3 years after complete remission in cases that take a very long time to achieve HBeAg seroconversion or if low HBV DNA loads persist after complete remission, even in young children (48).

An important factor affecting the probability of off-NA virological remission appears to be the duration of on-therapy HBV DNA undetectability (6). According to existing data, virological remission defined as HBV DNA <2,000–20,000 IU/ml is maintained in ~50% of such patients 3 years after stopping NAs if virological remission is maintained during therapy for >2 years (6).

According to the international guidelines established by the American Association for the Study of Liver Diseases (AASLD) and the European Association for the Study of the Liver (EASL), consolidation therapy should be maintained for at least one additional year after HBeAg seroconversion (6, 53), whereas three more years is mandatory according to the Asian Pacific Association for the Study of the Liver (APASL) guidelines (46, 54). Under selective pressure from NA treatment, mutant HBV can disseminate to other hepatocytes and replace wild-type HBV. Considering the life span of hepatocytes, it is important to inhibit the dissemination of HBV to adjacent non-infected hepatocytes, which may occur due to reactivation during regeneration (55).

### Monitoring After Complete Remission

The target of HBV treatment is inhibiting recurrence after achieving HBeAg seroconversion or, if possible, HBsAg seroconversion, as HBsAg seroconversion is not uncommon in young children ages 6–7 years (41).

Quantitative HBsAg may be a valuable marker for indicating the need to stop NA treatment (56). HBV RNA levels during treatment can predict HBeAg seroconversion (57). Serum HBV RNA levels correlate more strongly with intrahepatic cccDNA levels than HBV DNA load and HBsAg levels before and after treatment (58). The levels of hepatitis B core-related antigen (HBcrAg) and HBV RNA are also useful in predicting off-treatment relapse (59).

A combination of on-treatment quantitative HBeAg titer and its decline may be a good predictor of positive response to long-term NA treatment among patients with HBeAg-positive CHB. In particular, the combination of HBeAg titer and its decline at 24 weeks has been reported to strongly predict a 96-week virological response and HBeAg clearance (60).

The off-treatment HBsAg level is closely related to clinical relapse after treatment cessation. A serum HBsAg level of <2 log10 IU/ml is an excellent predictor of sustained off-treatment response in patients with CHB who have received ETV for a sufficient duration (61). Therefore, monitoring HBsAg levels at 3 years after starting NA treatment could be useful to predict treatment response (62).

### Prevention of Treatment Failure

First, treatment should not be considered in patients in the immune-tolerant phase. Initial ALT levels must be >2 × ULN to avoid resistance (46).

Second, if high ALT levels persist in a patient, other hepatic etiologies, such as reactive hepatitis associated with pneumonia or acute pyelonephritis, should be considered. For obese children with CHB, it may be impossible to identify whether the cause of ALT elevation is an active HBV infection or non-alcoholic fatty liver disease. Thus, if such patients fail to reduce their body weight, liver biopsies should be performed to determine the need to start antiviral treatment.

Third, poor drug compliance is another cause of treatment failure. The compliance of patients is of utmost importance to prevent the emergence of drug-resistant HBV. Physicians should educate patients with CHB about the importance of daily administration of medicines without omission.

Fourth, it is also important not to stop medication too early after achieving complete remission. An additional treatment period must be considered for >2–3 years after HBeAg seroconversion to prevent relapse (6, 46).

## Conclusion

Based on the understanding of translational medical research about viral replication, hepatocyte turnover, and cccDNA clearance, the therapeutic strategy for CHB could be modified.

Vertical transmission can be prevented by prenatal and postnatal prophylaxis. Treatment indications should be carefully evaluated in children with CHB. Timely treatment is better if initiated in the initial period of the immune-clearance phase. Physicians should update their knowledge on current hepatitis B treatment guidelines. Further, to improve therapeutic efficacy, the application of the strategies should be inspired by the results of translational medical research.

## Author Contributions

BK and B-HC: conceptualization, methodology, and writing—review and editing. All authors: data curation and writing—original draft. All authors contributed to the article and approved the submitted version.

## Funding

This work was supported by the National Research Foundation of Korea (NRF) grant funded by the Korean Government (MSIT; no. 2021R1A2C1011004).

## Conflict of Interest

The authors declare that the research was conducted in the absence of any commercial or financial relationships that could be construed as a potential conflict of interest.

## Publisher's Note

All claims expressed in this article are solely those of the authors and do not necessarily represent those of their affiliated organizations, or those of the publisher, the editors and the reviewers. Any product that may be evaluated in this article, or claim that may be made by its manufacturer, is not guaranteed or endorsed by the publisher.
